# Characteristic Changes in the Immune Landscape of Aging Liver: From Basic Mechanism to Clinical Transformation

**DOI:** 10.1155/jimr/6574389

**Published:** 2026-06-03

**Authors:** Tong Zhu, Aijing Xu, Wei Yin, Chengzhong Li

**Affiliations:** ^1^ Department of Infectious Diseases, Changhai Hospital, Naval Medical University, Shanghai, 200433, China, smmu.edu.cn

**Keywords:** age-related liver diseases, immune microenvironment, immunosenescence, inflammaging, liver aging

## Abstract

The global population is aging at an unprecedented rate, significantly increasing the burden of age‐related liver diseases. While the mechanisms of liver aging remain incompletely understood, immune senescence within the hepatic microenvironment has emerged as a pivotal, yet underexplored, contributing factor. With advancing age, the liver undergoes profound remodeling of its immune landscape, encompassing dysfunction in both innate and adaptive immunity. This immunosenescence heightens susceptibility to infectious liver diseases, accelerates the progression of metabolic liver conditions, increases the risk of drug‐induced liver injury (DILI), and promotes hepatocellular carcinoma (HCC) through compromised immune surveillance. In conclusion, aging‐driven reorganization of the hepatic immune microenvironment elevates disease risk and undermines treatment efficacy. Targeting these age‐related immune alterations therefore represents a promising therapeutic strategy for improving liver health in the elderly.

## 1. Introduction

An unprecedented demographic shift is leading to an older population. According to the U.S. Census Bureau’s An Aging World: 2015 [1], a rapid rise has been observed in the proportion of people aged 65 and over, not only in developed countries but especially in Asia and Latin America. The global population aged 65 and over will more than double from 617 million in 2015 to over 1.5 billion by 2050. By the late 2070 s, the global population aged 65 and older is projected to reach 2.2 billion, which surpasses the number of children under age 18 [2]. This profound demographic shift presents severe public health challenges, with the burden of age‐related liver diseases growing increasingly The incidence, complexity, and treatment difficulty of viral hepatitis (e.g., hepatitis B virus/hepatitis C virus [HBV/HCV]), alcoholic liver disease, metabolic dysfunction‐associated steatotic liver disease (MASLD) and its progression form, metabolic dysfunction‐associated steatohepatitis (MASH), drug‐induced liver injury (DILI), hepatic fibrosis/cirrhosis, and hepatocellular carcinoma (HCC) have all substantially increased among older adults [3, 4]. To date, the mechanisms underlying liver aging remain unclear. Comprehending the unique pathophysiological mechanisms driving these diseases in elderly populations has become an urgent need in hepatology and geriatric medicine, with liver immune senescence serving as a core yet underexplored factor.

The liver serves not only as a vital organ for metabolism and detoxification but also as a highly specialized frontline immune checkpoint. Exposed to a rich stream of intestinal antigens, including nutrients, symbiotic bacteria, and potential pathogens, it hosts diverse immune cell populations: resident macrophages (Kupffer cells, [KCs]), liver sinusoidal endothelial cells (LSECs), hepatic stellate cells (HSCs), and abundant lymphocyte subsets (T‐lymphocyte cells, B‐lymphocyte cells, and natural killer [NK] cells) [5]. These cells collectively form an intricate immune surveillance network that contributes to the maintenance of tolerance and defense within the gut–liver axis. However, similar to systemic immune senescence, the liver’s immune system undergoes profound age‐related changes. Different from the mere replication of systemic aging, these alterations are determined by the liver’s unique microenvironment, which profoundly influences its physiological functions, disease susceptibility, and therapeutic responses.

Here, we review age‐related changes in the liver that have substantial biological and clinical consequences for older adults.

## 2. Physiological and Immunological Microenvironment of Aging Liver

### 2.1. Age‐Related Structural and Cellular Changes in the Liver

Liver aging is characterized by macroscopic, microscopic, and cellular alterations that set the stage for microenvironmental dysfunction. Macroscopically, both liver volume and hepatic blood flow declined significantly with advancing age. Studies indicate that liver volume can decrease by 20%–40% in older adults, with sex‐specific patterns: a significant reduction becomes evident in men after 65 years of age, whereas in women, it begins between 40 and 64 years and progresses further thereafter. Hepatic blood flow also declines by ~35%–50% with age, a reduction that generally corresponds to the decrease in liver volume [5–8]. Autopsy studies show a concomitant decrease in liver weight by 24% in men and 18% in women [9]. At the parenchymal level, aging manifests through a reduction in the hepatocyte number and a notable decline in regenerative capacity [5]. These hepatocytes exhibit impaired autophagy and increased oxidative stress, leading to an elevated binuclear hepatocyte index, decreased smooth endoplasmic reticulum concentration, and reduced microsomal enzyme activity (e.g., monooxygenases and glucose‐6‐phosphatase) [10]. Consequently, apoptotic, senescent, and polyploid hepatocytes accumulate in the aged liver [11, 12].

Beyond hepatocytes, the biliary epithelium undergoes significant age‐related remodeling. Cholangiocytes, the epithelial cells lining the bile ducts, acquire a senescent phenotype marked by telomere shortening, cell‐cycle arrest, and the development of a proinflammatory senescence‐associated secretory phenotype (SASP) [13]. This premature senescence can be triggered by various stressors prevalent in aging and liver disease, including oxidative stress, endoplasmic reticulum stress, and dysregulated signaling pathways such as the secretin/secretin receptor axis [14]. In aging and chronic cholestatic liver diseases (e.g., primary biliary cholangitis), senescent cholangiocytes demonstrate bile duct hyperplasia, upregulation of senescence markers (e.g., p16 and p21) and proteins like twinfilin 1, and secretion of a spectrum of SASP factors including IL‐6 and IL‐8, CCL2, and TGF‐β1 [5, 14–17]. At the subcellular level, aging cholangiocytes exhibit reduced autophagy, which may contribute to their dysfunction and SASP amplification [5]. Importantly, the senescence state of cholangiocytes dynamically interacts with that of HSCs, creating a regulatory axis that influences fibrogenic progression [18]. Functionally, these changes are associated with reduced bile flow [5]. Furthermore, the anatomical and functional coupling between the biliary tract and its surrounding peribiliary vascular plexus may also be compromised with age, potentially disrupting nutrient supply, signal exchange, and the reuptake of biliary components [19].

Structurally, the hepatic sinusoid, the primary site for blood‐hepatocyte exchange, undergoes a profound transformation with aging, a process central to understanding the altered liver microenvironment. Key among these changes is the pseudocapillarization of LSECs, which involves the loss of fenestrations, basement membrane thickening, and extracellular matrix (ECM) deposition [20–23]. This transformation creates a diffusion barrier that not only impedes the hepatic uptake of substrates (e.g., chylomicron remnants and insulin) but also severely hinders the sinusoidal patrolling of immune cells like T cells and neutrophils, thereby compromising immune surveillance and the clearance of gut‐derived antigens [21, 24–26].

### 2.2. Molecular Landscapes of the Aged Hepatic Immune Microenvironment

Cellular changes in aged human liver are mechanistically grounded in molecular alterations revealed by multiomics studies in model systems. Integrated spatial, single‐cell, and metabolomic profiling in the aging mouse liver demonstrates zonation‐specific decay: periportal hepatocytes exhibit mitochondrial dysfunction and altered lipid metabolism, whereas pericentral hepatocytes upregulate lipid‐droplet genes (e.g., Cidea/Cidec) and accumulate large lipid droplets. Epigenetically, chromatin accessibility shifts in a zonation‐dependent manner, with aging‐linked topics enriched for metabolic pathways and showing transcriptional–epigenetic decoupling [27]. These intrinsic changes converge with immune–stromal reprograming. In rodents, scRNA‐seq reveals expansion of inflammatory macrophages and neutrophils, alongside profibrotic activation of stellate cells and sinusoidal endothelial dysfunction [28]. Primate atlases further highlight zonated SREBP2 hyperactivation in hepatocytes, driving senescence and metabolic decline [29]. Notably, a specialized subset of CXCL2+ macrophages identified in mice exacerbates tissue injury via neutrophil recruitment and neutrophil extracellular trap (NET) formation, a mechanism conserved in human liver samples [30]. Collectively, multiomics insights from animal models depict a reinforcing cycle: metabolic–epigenetic stress in hepatocytes synergizes with inflammatory activation of immune and stromal cells, disrupting homeostasis and priming the liver for fibrosis and injury. These findings provide a mechanistic framework for understanding the immune microenvironment in the aging human liver.

## 3. Specific Effects of Aging on Liver Immune System

**Figure 1:**
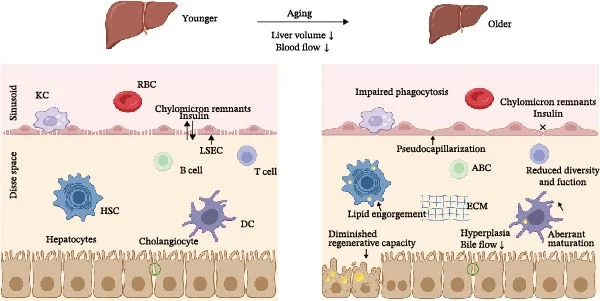
Alterations in the aging liver. With aging, the liver undergoes a series of alterations in physiological and immunological microenvironments. Liver volume and hepatic blood flow decrease. Hepatocytes exhibit a reduced population, diminished regenerative capacity, and an increased binuclear index. The immune function is compromised, characterized by declined phagocytic capacity of KCs, dysmaturity of DCs, reduced diversity and function of T cells, and the accumulation of ABCs. Additionally, HSCs become engorged with lipid droplets and drive ECM deposition, while LSECs undergo pseudocapillarization. ABC, age‐associated B cells; DC, dendritic cell; ECM, extracellular matrix; HSC, hepatic stellate cell; KC, Kupffer cell; LSEC, liver sinusoidal endothelial cell; RBC,; red blood cell.

Aging induces not only an overall structural and functional decline in the liver but also a progressive deterioration of its local immune surveillance and regulatory capacity, ultimately impacting systemic immune homeostasis. These age‐related alterations are primarily characterized by dysfunction in both innate and adaptive immune cells, alongside structural and functional abnormalities in key nonparenchymal cells, particularly HSCs and LSECs. The major changes across these cell types are summarized in Table 1 and Figure 1.

**Table 1:** Age‐related alterations in key hepatic cell populations.

Cell type	Young liver features	Changes in aging liver	Core mechanism
KC	• Sentinel cells [31, 32] • Effective phagocytosis [31, 32]	• Increased population [31, 32] • Impaired phagocytosis [31, 32] • Proinflammatory activation [13, 31–34] • Increased infiltration of monocyte‐derived macrophages (M1‐like polarization) [35]	• Mitochondrial dysfunction: ROS/NLRP3 inflammasome activation [36].
DC	• Balances immune tolerance and immunogenicity [3]	• Numerical increase with skewed subsets [37] • Lipid accumulation and semi‐activated state in CD4‐CD8α+ DCs [37] • Aberrant maturation: loss of tolerance balance [3, 38]	• Inflammatory milieu [37] • Intrinsic maturation defects [38]
T cell	• Thymic‐derived [39] • Robust function and diversity [39] • Effective surveillance [39]	• Extrathymic origins [39, 40] • Phenotypically distinct from blood [39] • Reduced diversity and function [41] • Suppressive microenvironment [41]	• Thymic involution [39] • Inflammaging‐induced dysfunction and Treg expansion [41] • Altered antigen recognition leading to autoreactivity [39, 42]
B cell	• Diverse repertoire [43] • Functional antibody responses [44, 45]	• Repertoire collapse [43] • Impaired antibody responses [44, 45] • Expansion of ABCs [46] • ABCs secrete proinflammatory cytokines and drive T‐cell senescence [46]	• Hyperinsulinemia: PI3K/AKT/mTOR axis activation [47]
HSC	• Quiescent state [5] • Maintains sinusoidal architecture [5]	• Activation and lipid engorgement [48–51] • Fibrosis promotion [48, 52] • ECM deposition [49]	• Profibrogenic activation [5] • Altered liver microstructure [5]
LSEC	• Fenestrated endothelium [23] • Efficient substrate transfer [26]	• Pseudocapillarization [53] • Impaired endocytosis [53, 54]	• Structural remodeling [53] • Basal lamina deposition [53, 54]

Abbreviations: ABCs, age‐associated B cells; DC, dendritic cell; ECM, extracellular matrix; HSC, hepatic stellate cell; KC, Kupffer cell; LSEC, liver sinusoidal endothelial cell.

### 3.1. Innate Immune Cell Senescence

KCs, the liver’s resident macrophages, act as sentinel cells by phagocytosing blood‐borne pathogens, cellular debris, and toxins. Studies in rodent models indicate that during liver aging, the population of KCs expands while their phagocytic capacity declines [31, 32]. These cells enter a state of low‐grade, yet dysfunctional, activation [32, 33]. This altered state is a major driver of hepatic “inflammaging”, characterized by elevated expressions of proinflammatory mediators, including tumor necrosis factor (TNF), and diminished expression of anti‐inflammatory cytokines such as interleukin (IL)−10 [13, 31, 34]. Furthermore, this proinflammatory shift is coupled with features of immunosenescence, manifesting as a further dampened phagocytic response to particulate stimuli and a blunted acute‐phase response to infections [32, 55]. Consequently, aged KCs are less effective at clearing pathogens and debris while simultaneously promoting a chronic inflammatory state within the liver. Mitochondrial dysfunction is one of the core mechanisms underpinning this dysfunctional shift. In senescent KCs, weakened oxidative phosphorylation capacity leads to reactive oxygen species (ROS) accumulation that activates NOD‐, LRR‐, and pyrin domain‐containing protein 3 (NLRP3) inflammasomes, which amplifies inflammatory cascades and impairs antigen presentation functions [36].

In addition to KCs, monocyte‐derived macrophages (also referred to as infiltrating macrophages) play a significant role in aging‐related liver changes. These macrophages originate from circulating monocytes and are recruited to the liver during inflammation or injury. In aged livers, increased infiltration of monocyte‐derived macrophages is observed, which contributes to chronic low‐grade inflammation and fibrosis [35]. Unlike KCs, which are primarily self‐renewing, monocyte‐derived macrophages in aged livers often exhibit enhanced proinflammatory polarization (M1‐like) and impaired transition to reparative (M2‐like) phenotypes, thereby exacerbating tissue damage and delaying resolution of inflammation [31, 35].

Dendritic cells (DCs) are specialized antigen‐presenting cells crucial for immune surveillance. Immature DCs promote tolerance through the induction of regulatory T cells (Tregs), whereas mature DCs stimulate effector T cells [3]. Mechanistic insights from aging mouse models reveal profound changes in the hepatic DC compartment. DCs increase in number and exhibit a skewed subpopulation profile, marked by the accumulation of plasmacytoid DCs (pDCs) and the CD4‐CD8α+ DCs subset. These CD4‐CD8α+ DCs accumulate lipids and adopt a semi‐activated state, a phenotype associated with functional dysregulation that contributes to inflammaging while impairing adaptive immunity [37]. These age‐related alterations likely underpin the compromised DC function observed in the elderly. In humans, DCs exhibit inappropriate maturation induced by infections or tissue injury, which can alter the balance between their tolerogenic and immunogenic functions and may trigger the development of autoimmune diseases [3, 38]. Interestingly, long‐term administration of *Lactococcus lactis*‐derived plasma activates pDCs, which helps in maintaining immune function and potentially decelerates aging while extending lifespan [56].

### 3.2. Adaptive Immune Cell Senescence

Studies on T lymphocytes in mice indicate that the population of intermediate T‐cell receptor (TCR(int)) cells, which includes Natural Killer T (NKT) cells, increases until middle age but subsequently declines, impairing a critical first‐line defense against invading tumor cells [39, 57]. Furthermore, the pathway for T‐cell development shifts with age: extrathymic T‐cell differentiation, which is of minor importance in normal young mice, becomes predominant in aged mice [39, 40]. These extrathymically derived T cells undergo less stringent selection in the liver, leading to a population with more pronounced autoreactive characteristics [39, 42]. Notably, hepatic T cells are phenotypically distinct from their counterparts in peripheral blood. They exhibit a reversed CD4:CD8 ratio, a higher frequency of TCRγδ + cells, and unique subsets such as CD8α+CD8β–T cells, reflecting the liver’s unique immune microenvironment [39]. These phenotypic features are functional correlates of the liver’s extrathymic differentiation pathway and its tolerogenic milieu, conferring innate‐like responsiveness and altered antigen recognition. This may underlie the enhanced autoreactive potential of the aged hepatic T‐cell pool [39]. This shift, potentially influenced by age‐related hormonal changes such as estrogen fluctuations, may contribute to the increased prevalence of autoimmunity and altered immune surveillance in the aging liver [39, 58]. Additionally, aging is associated with a general decline in T‐cell function, including reduced infiltration of CD4+ and CD8+ T cells into tissues, impaired cytotoxic activity, and an increase in Tregs, which collectively suppress antitumor immunity and promote a tolerogenic microenvironment [41].

B lymphocytes are essential for hepatic immune surveillance and tolerance, but their dysregulation can exacerbate fibrosis and chronic liver disease [59]. During aging, the hepatic B‐cell compartment undergoes profound remodeling. At the population level, this is characterized by a marked decline in B‐cell receptor diversity and a collapse of repertoire breadth, as demonstrated by spectratyping and sequencing studies [43]. At the functional level, aged B cells exhibit intrinsic defects, including downregulation of the transcription factor E2A (E47) and its target activation‐induced cytidine deaminase (AID), leading to impaired immunoglobulin class switch recombination and reduced production of high‐affinity antibodies [44, 45]. Concurrently, there is a notable expansion of a unique, age‐associated B‐cell subset (ABCs, defined as CD21‐CD23‐CD11c+T‐bet+ in mice and IgD‐CD27‐CD11c+T‐bet+ in humans) [46]. Critically, ABCs are not merely biomarkers of aging but active drivers of immunosenescence. They secrete proinflammatory cytokines (e.g., IFNγ, TNF) and act as potent antigen‐presenting cells, engaging in pathogenic crosstalk that depletes naive T cells, promotes exhausted/senescent T‐cell phenotypes, and reduces T‐cell receptor diversity [46]. Mechanistically, age‐related hyperinsulinemia fuels ABC expansion and inflammatory output via the insulin receptor–PI3K/AKT/mTOR axis in B cells. Human cohort data corroborate this link, showing that peripheral ABC abundance correlates with inflammatory aging (iAge) and T‐cell senescence signatures [47]. Thus, aging‐induced B cell defects and ABC expansion jointly compromise antibody responses and promote T‑cell senescence through chronic inflammation, leading to a self‑propagating state of immune dysregulation in the aging liver.

### 3.3. Nonimmune Cell‐Related Senescence

HSCs play a primarily indirect role in age‐related immune changes, that is, through their central role in promoting fibrosis, which alters the liver’s architecture and microenvironment. Although the exact changes in the HSC number or activation state with aging are still being defined [48, 60], their role in perisinusoidal fibrosis is well‐established. This fibrosis is likely secondary to either increased collagen production by activated HSCs or impaired collagen clearance by LSECs [48, 52]. Studies in rodents and nonhuman primates show that HSCs become engorged with large lipid droplets that protrude into the sinusoid [48–51]. This physical distortion, along with the deposition of the ECM, increases vascular resistance and impedes sinusoidal blood flow [49]. These altered hemodynamics and fibrosis contribute to a proinflammatory and profibrotic microenvironment, disrupting normal liver physiology.

LSECs undergo a defined senescent program termed “pseudocapillarization” [53]. In addition to the structural changes described above, aged LSECs exhibit functional senescence: their scavenger endocytic capacity is markedly reduced, while the expression of adhesion molecules (e.g., ICAM‐1) is increased [53, 54]. This functionally senescent phenotype enhances leukocyte adhesion within sinusoids, further reducing perfusion and impairing the systemic clearance of waste macromolecules [53, 54]. Importantly, this process is reversible; interventions like caloric restriction can attenuate these changes, highlighting LSEC senescence as a therapeutic target [54].

### 3.4. Dysregulated Cellular Crosstalk in the Aging Liver Microenvironment

The age‐related alterations in individual cell types, detailed in the preceding sections, do not occur in isolation. Instead, they collectively disrupt the intricate network of cellular communication, generating a self‐amplifying pathological cycle, as evidenced by studies in animal models. In rodent livers, the SASP secreted by senescent hepatocytes actively recruits and activates resident immune cells, such as KCs and monocyte‐derived macrophages, and directly activates HSCs [61]. In turn, the aged immune compartment amplifies parenchymal damage. A key example is the expansion of a specialized subset of CXCL2+ macrophages within the inflammatory macrophage pool of the aged mouse liver. These cells recruit large numbers of neutrophils via the CXCL2‐CXCR2 axis, directly exacerbating tissue injury [30]. Recruited neutrophils further propagate the senescent niche by inducing paracrine senescence in neighboring hepatocytes through ROS‐mediated telomeric DNA damage [62]. Furthermore, macrophage‐derived TGFβ has been identified in mice as a critical mediator that propagates senescence between hepatocytes during acute injury, thereby suppressing liver regeneration [63]. Simultaneously, activated HSCs, beyond their role in ECM deposition, secrete chemokines that promote excessive neutrophil infiltration and ROS production. These factors, in turn, inhibit the activation and proliferative capacity of liver progenitor cells, thereby compromising tissue repair [64]. Concurrently, the age‐related pseudocapillarization of LSECs creates a physical barrier that impedes the sinusoidal patrolling and extravasation of effector lymphocytes and NK cells. This functionally excludes them from sites of early injury or transformation, compromising immunosurveillance [21, 24]. Collectively, this triangular dysregulation remodels the aged hepatic microenvironment into a proinflammatory, profibrotic, and immunosuppressive milieu. This remodeled niche underlies the increased severity of metabolic liver disease, accelerated fibrogenesis, and diminished antitumor immunity characteristic of the aging liver.

## 4. Clinical Significance of Liver Immune Senescence

The remodeling of the hepatic immune microenvironment with age, as described above, fundamentally reshapes the liver’s response to injury and disease, translating into distinct clinical manifestations and outcomes in the elderly. Against this backdrop of underlying immunosenescence, routine clinical assessments present a complex picture.

Routine liver function tests typically remain within normal limits in healthy older adults, which suggests substantial resilience of hepatic physiological function to aging [5, 65]. Notably, alanine transaminase (ALT) activity exhibits an age‐related decline, with low serum ALT levels in older adults emerging as a risk factor for all‐cause mortality and frailty [66]. Conversely, elevations of liver function test results beyond normal limits in the elderly (prevalence ~ 16% in those ≥75 years) signify underlying liver disease or comorbid conditions such as stroke, hypertension, dementia, or cancer [33]. Critically, elevations in two or more liver function tests are associated with a twofold increase in overall mortality and a 17‐fold increase in liver‐specific mortality. Elevated liver function tests in older individuals correlate more strongly with comorbid diseases and overall mortality than with primary liver pathology [67].

### 4.1. Infectious Liver Disease: The Vicious Cycle of Immune Senescence

The aged liver enters a state of vulnerability due to its preexisting senescent and proinflammatory immune microenvironment, characterized by dysfunctional KCs and inflammaging. Chronic infections (e.g., HCV, HBV, or cytomegalovirus) act as a powerful “second hit,” which considerably exacerbates this immune dysfunction. The persistent exposure to pathogen‐associated molecular patterns (PAMPs) delivers sustained, high‐intensity activation signals to the already dysregulated KCs and other innate immune cells. This leads to exaggerated activation of the NLRP3 inflammasome, profound mitochondrial dysfunction, and increased oxidative stress, which collectively impair hepatocyte regeneration and repair [68–70]. Moreover, these infections further disrupt adaptive immunity by promoting antigen‐specific T‐cell clonal expansion and subsequent exhaustion, filling the immune repertoire with terminally differentiated effector/memory cells. Coupled with the intrinsic decline in immune surveillance and weakened vaccine responses in the aged liver, these changes create a vicious cycle: the infection amplifies age‐related immunosenescence while the senescent immune system fails to achieve effective viral clearance. This results in significantly diminished pathogen clearance capacity and accelerated fibrotic progression, as evidenced by the more severe disease course observed in elderly patients with chronic hepatitis [68]. These changes collectively increase older individuals’ susceptibility to infectious liver diseases and the risk of disease progression.

### 4.2. Noninfectious Liver Disease

MASLD constitutes a major global public health challenge, affecting ~24% of the world’s adult population. Its epidemiology is closely tied to age, with prevalence rising through adulthood and peaking in middle age, specifically between 40–50 years in males and 60–69 years in females [71]. Interestingly, epidemiological studies describe an “inverted U‐shaped” curve, where prevalence appears to plateau or slightly decline in the very elderly (>70 years) [72]. Importantly, despite a potential prevalence dip in advanced age, elderly patients present with a higher burden of cardiometabolic risk factors and are at significantly increased risk for progressive diseases, including advanced fibrosis and HCC [71]. Molecular studies, primarily in cellular and animal models, have identified key mechanisms driving hepatic lipid accumulation. These include increased ROS formation and DNA damage [73], telomere shortening [73], decreased autophagy [74], activation of lipogenic pathways such as p300‐C/EBP‐dependent neutral fat synthesis [75], and inflammatory activation mediated by M1‐like macrophages and nuclear factor (NF)‐*κ*B pathways [60]. Emerging evidence suggests that metabolic dysfunction itself can be a direct inducer of hepatocyte senescence. A knock‐in mouse model mimicking constitutive acetylation of mitochondrial acetyl‐CoA synthetase 1 at lysine 635 exhibited disrupted hepatic energy metabolism (evidenced by decreased ATP and elevated acetate) and aberrant lipid homeostasis. This metabolic perturbation predisposes the liver to rapid steatosis upon stress and, crucially, leads to a marked upregulation of senescence markers (e.g., p21 and SA‐*β*‐gal) in hepatocytes even under basal conditions. In turn, these senescent cells can aggravate disease progression via the SASP, which promotes chronic inflammation, recruits immune cells, and alters the ECM to foster a profibrotic microenvironment [61]. Therefore, a self‐perpetuating cycle may exist in the aging liver: inherent metabolic vulnerabilities drive cellular senescence, and senescent cells actively contribute to inflammation and fibrosis through paracrine signaling. This mechanism likely underlies the increased severity of MASLD and MASH in older individuals.

DILI is a significant clinical concern in the elderly, where age‐related physiological decline intersects with polypharmacy. Quantitative liver function tests using 99mTc‐mebrofenin or 99mTc‐galactosyl human serum albumin scintigraphy have revealed a linear reduction in liver clearance of 20%–40% in healthy humans with age [5, 76, 77]. The reduction in drug metabolism is secondary to age‐related decreases in hepatic blood flow, liver volume, and enzyme activity [78]. This condition leads to reduced drug clearance rates and prolonged half‐lives, which substantially increase the risk of DILI in elderly patients [79, 80]. Although advanced age may not be a general risk factor for DILI incidence, it profoundly influences the DILI phenotype, which favors a cholestatic pattern of injury [81, 82]. Mechanistically, aging can reduce the ATP supply and the activity of key transport proteins. This compromises the liver’s ability to compensate for additional insults, such as drug‐induced inhibition of the bile salt export pump (BSEP), thereby enhancing bile acid accumulation in the hepatocytes and leading to cellular damage [82]. Furthermore, this phenotypic shift is underpinned by age‐related alterations in the immune system. Aging, through immunosenescence and inflammaging, lowers the threshold for breaking hepatic immune tolerance, thereby facilitating an immune response against the liver under the stress of drug exposure [82–85]. Moreover, aging is accompanied by alterations in the gut microbiota composition and increased intestinal permeability [86, 87], which potentially lead to the translocation of gut‐derived PAMPs (e.g., lipopolysaccharide) into the circulation. This condition can further prime and activate the innate immune system in the liver, which creates a permissive environment for DILI initiation [85]. Thus, while older adults may not be inherently more susceptible to triggering DILI, their aged hepatic milieu, characterized by diminished metabolic and excretory reserve, a proinflammatory immune microenvironment, and a compromised gut barrier, collectively favors the emergence of a specific cholestatic DILI phenotype. In addition, the high burden of comorbidities in this population contributes to increased nonliver‐related mortality following DILI [82, 88].

### 4.3. Liver Tumors: Age‐Related Loss of Immune Surveillance

HCC can be considered a liver disease that is characteristic of older age because most adults who are diagnosed with HCC are 70 years of age or older [89]. However, the incidence of HCC decreases in people over 85 years of age [5, 90]. The aged liver fosters a uniquely permissive microenvironment for HCC through the fundamental hallmarks of aging. At the cellular and molecular levels, processes such as genomic instability, telomere attrition, epigenetic alterations, and cellular senescence directly overlap with the hallmarks of cancer, creating a core biological link between advancing age and increased cancer risk [5, 89]. The resulting state of inflammaging, driven largely by the SASP, disrupts normal immune surveillance through pathways such as NF‐*κ*B and promotes an immunosuppressive and protumorigenic microenvironment [91, 92]. Therefore, the aged liver does not merely coincide with HCC; it actively establishes a proinflammatory, functionally permissive, and structurally remodeled niche that fundamentally enables the initiation and progression of HCC in older patients.

## 5. Summary and Outlook

In summary, the aging liver undergoes a profound remodeling of its immune microenvironment, marked by structural changes, functional declines in both innate and adaptive immunity, and a state of inflammaging. These alterations collectively compromise immune surveillance, promote autoreactivity, accelerate fibrosis, and increase susceptibility to infections, metabolic liver disease, and HCC.

Given these profound alterations, the mechanisms of aging itself emerge as a common pathogenic driver across various liver diseases. Directly targeting these aging‐related alterations offers a novel therapeutic rationale. For instance, in MASLD/MASH, which is driven by senescent hepatocytes leading to fat accumulation, inflammation, and fibrosis, the use of senolytics to clear senescent cells or nutritional interventions such as caloric restriction to improve metabolism aims to block the core disease process at its source [68, 93]. For DILI, which is more prevalent in the elderly and often presents with a cholestatic pattern, intervention strategies focus on restoring the age‐related decline in drug metabolism and transport functions. This can be achieved through pharmacological or nutritional means to improve sinusoidal blood flow and cellular detoxification capacity, thereby preventing bile acid accumulation and hepatocyte injury [5, 68]. In the prevention and treatment of HCC, targeting the unique inflammaging microenvironment and the decline in immune surveillance in the aged liver is crucial. Strategies include modulating immunosenescence (e.g., by improving mitochondrial function and inhibiting the SASP) or developing immune checkpoint modulators tailored to the aged immune state to remodel the tumor microenvironment and enhance the efficacy of immunotherapy [5, 68]. Therefore, targeting the fundamental biology of liver aging offers a promising unifying strategy for the prevention and treatment of multiple age‐related liver diseases.

## Author Contributions

**Tong Zhu**: conceptualization, writing – review and editing, writing – original draft. **Aijing Xu**: conceptualization, writing – review and editing. **Wei Yin**: figure preparation. **Chengzhong Li**: read and edited the final manuscript.

## Funding

The authors declare that no financial support was received for the research and/or publication of this article.

## Disclosure

All authors have read and agreed to the published version of the manuscript.

## Conflicts of Interest

The authors declare no conflicts of interest.

## Data Availability

Data sharing is not applicable to this article as no datasets were generated or analyzed during the current study.

## References

[1] He W. , Goodkind D. , and Kowal P. , An Aging World:2015, 2016.

[2] Global Issues: Ageing, https://www.un.org/en/global-issues/ageing.

[3] Kazuto T. and Yukihiro S. , Liver Physiology and Liver Diseases in the Elderly, World Journal of GastroenterologyWorld Journal of Gastroenterology. (2014) 19, no. 46. 10.3748/wjg.v19.i46.8459 PMC3870491 24379563

[4] Rinella M. E. , Lazarus J. V. , and Ratziu V. , et al.A Multisociety Delphi Consensus Statement on New Fatty Liver Disease Nomenclature, Journal of Hepatology. (2023) 78, no. 6. 10.1097/HEP.0000000000000520 PMC10653297 37363821

[5] Le Couteur D. G. , Ngu M. C. , Hunt N. J. , Brandon A. E. , Simpson S. J. , and Cogger V. C. , Liver, Ageing and Disease, Nature Reviews Gastroenterology & Hepatology. (2025) 22, no. 10, 680–695, 10.1038/s41575-025-01099-z. 40721658

[6] Wynne H. A. , Cope L. H. , Mutch E. , Rawlins M. D. , Woodhouse K. W. , and James O. F. W. , The Effect of Age Upon Liver Volume and Apparent Liver Blood Flow in Healthy Man, Hepatology. (1989) 9, no. 2, 297–301, 10.1002/hep.1840090222. 2643548

[7] Zeeh J. and Platt D. , The Aging Liver: Structural and Functional Changes and Their Consequences for Drug Treatment in Old Age, Gerontology. (2002) 48, no. 3, 121–127, 10.1159/000052829. 11961363

[8] Sanfeliu-Redondo D. , Gibert-Ramos A. , and Gracia-Sancho J. , Cell Senescence in Liver Diseases: Pathological Mechanism and Theranostic Opportunity, Nature Reviews Gastroenterology & Hepatology. (2024) 21, no. 7. 10.1038/s41575-024-00913-4 38485755

[9] Zeeh J. , The Aging Liver: Structural and Functional Changes and Their Consequences for Drug Treatment in Old Age, Gerontologia. (2002) 48, no. 3. 10.1159/000052829 11961363

[10] Yalei Z. , Ya Y. , Qian L. , and Jianzhou L. , Understanding the Unique Microenvironment in the Aging Liver, Frontiers in Medicine. (2022) 9. 10.3389/fmed.2022.842024 PMC8907916 35280864

[11] Hua-Hua Z. , Shao-Jie H. , and Bo Y. , et al.Apoptosis in the Aging Liver, Oncotarget. (2017) 8, no. 60. 10.18632/oncotarget.21123 PMC5731987 29254277

[12] Min-Jun W. , Fei C. , and Jian-Xiu L. , et al.Reversal of Hepatocyte Senescence After Continuous In Vivo Cell Proliferation, Hepatology. (2014) 60, no. 1. 10.1002/hep.27094 24711261

[13] Leonardo B. , Shannon G. , and Heather F. , et al.Impact of Aging on Liver Cells and Liver Disease: Focus on the Biliary and Vascular Compartments, Hepatology Communications. (2021) 5, no. 7. 10.1002/hep4.1725 PMC8279468 34278165

[14] Meadows V. , Baiocchi L. , and Kundu D. , et al.Biliary Epithelial Senescence in Liver Disease: There Will Be SASP, Frontiers in Molecular Biosciences. (2021) 8, 10.3389/fmolb.2021.803098. PMC8724525 34993234

[15] Motoko S. , Yasunori S. , and Yasuni N. , Increased p16(INK4a)-Expressing Senescent Bile Ductular Cells Are Associated With Inadequate Response to Ursodeoxycholic Acid in Primary Biliary Cholangitis, Journal of Autoimmunity. (2019) 107, 102377. 31812332 10.1016/j.jaut.2019.102377

[16] Gaudio E. , Onori P. , Franchitto A. , Pannarale L. , Alpini G. , and Alvaro D. , Hepatic Microcirculation and Cholangiocyte Physiopathology, Italian Journal of Anatomy and Embryology. (2005) 110, 71–5. 16101023

[17] Motoko S. , Hiroko I. , Junpei Y. , Satoko N. , and Yasuni N. , Telomere Shortening in the Damaged Small Bile Ducts in Primary Biliary Cirrhosis Reflects Ongoing Cellular Senescence, Hepatology. (2008) 48, no. 1. 10.1002/hep.22348 18536059

[18] Ying W. , Fanyin M. , and Nan W. , et al.Substance P Increases Liver Fibrosis by Differential Changes in Senescence of Cholangiocytes and Hepatic Stellate Cells, Hepatology. (2017) 66, no. 2. 10.1002/hep.29138 PMC5519428 28256736

[19] Gaudio E. , Onori P. , Pannarale L. , and Alvaro D. , Hepatic Microcirculation and Peribiliary Plexus in Experimental Biliary Cirrhosis: A Morphological Study, Gastroenterology. (1996) 111, no. 4, 1118–1124, 10.1016/s0016-5085(96)70081-1. 8831608

[20] Yan L. , Qiao L. , and Guangyu L. , et al.Overview of Innate Immune Cell Landscape in Liver Aging, International Journal of Molecular Sciences. (2024) 25, no. 1. 10.3390/ijms25010181 PMC10778796 38203352

[21] Couteur D. G. L. , Warren A. , and Cogger V. C. , et al.Old Age and the Hepatic Sinusoid, The Anatomical Record. (2008) 291, no. 6. 10.1002/ar.20661 18484614

[22] Mak K. M. , Chu E. , Lau K. H. V. , and Kwong A. J. , Liver Fibrosis in Elderly Cadavers: Localization of Collagen Types I, III, and IV, α-Smooth Muscle Actin, and Elastic Fibers, The Anatomical Record. (2012) 295, no. 7. 10.1002/ar.22504 22644959

[23] Johanne P. , Sara L. , and Chantal B. , et al.Liver Sinusoidal Endothelial Cells: Physiology and Role in Liver Diseases, Journal of Hepatology. (2016) 66, no. 1. 10.1016/j.jhep.2016.07.009 27423426

[24] Warren A. , Le Couteur D. G. , Fraser R. , Bowen D. G. , McCaughan G. W. , and Bertolino P. , T Lymphocytes Interact With Hepatocytes Through Fenestrations in Murine Liver Sinusoidal Endothelial Cells, Hepatology. (2006) 44, no. 5. 10.1002/hep.21378 17058232

[25] Hilmer S. N. , Cogger V. C. , Fraser R. , McLean A. J. , Sullivan D. , and Le Couteur D. G. , Age-Related Changes in the Hepatic Sinusoidal Endothelium Impede Lipoprotein Transfer in the Rat, Hepatology. (2005) 42, no. 6. 10.1002/hep.20937 16317689

[26] Mashani M. , Sarah Jayne M. , and Lindsay Edward W. , et al.Ultrastructure of the Liver Microcirculation Influences Hepatic and Systemic Insulin Activity and Provides a Mechanism for Age-Related Insulin Resistance, Aging Cell. (2016) 15, no. 4. 10.1111/acel.12481 PMC4933657 27095270

[27] Chrysa N. , Niklas K. , and Swati P. , et al.Spatial and Single-Cell Profiling of the Metabolome, Transcriptome and Epigenome of the Aging Mouse Liver, Nature Aging. (2023) 3, 1430–1445. 37946043 10.1038/s43587-023-00513-y PMC10645594

[28] Yan L. , Ying L. , and Guangyu L. , et al.Single-Cell Transcriptome Analysis of Aging Mouse Liver, Faseb Journal. (2024) 38, no. 4. 10.1096/fj.202302282R 38334462

[29] Shanshan Y. , Chengyu L. , and Mengmeng J. , et al.A Single-Nucleus Transcriptomic Atlas of Primate Liver Aging Uncovers the Pro-Senescence Role of SREBP2 in Hepatocytes, Protein & Cell. (2023) 15, no. 2. 10.1093/procel/pwad039 PMC10833472 37378670

[30] Yasong L. , Jiaqi X. , and Jianye C. , et al.Single-Cell Immune Profiling of Mouse Liver Aging Reveals Cxcl2+ Macrophages Recruit Neutrophils to Aggravate Liver Injury, Hepatology. (2023) 79, no. 3. 10.1097/HEP.0000000000000590 PMC10871588 37695548

[31] Stranks A. J. , Hansen A. L. , and Panse I. , et al.Autophagy Controls Acquisition of Aging Features in Macrophages, Journal of Innate Immunity. (2015) 7, no. 4. 10.1159/000370112 PMC4386145 25764971

[32] Hilmer S. N. , Cogger V. C. , and Le Couteur D. G. , Basal Activity of Kupffer Cells Increases With Old Age, The Journals of Gerontology. Series A, Biological Sciences and Medical Sciences. (2007) 62, no. 9, 973–8, 10.1093/gerona/62.9.973. 17895435

[33] Cristina M. , Maria Giulia B. , and Aurelia S. , et al.The Peculiar Aging of Human Liver: A Geroscience Perspective Within Transplant Context, Ageing Research Reviews. (2019) 51. 10.1016/j.arr.2019.02.002 30772626

[34] David C. R. , Guillermo M. , and Guido K. , Autophagy and Aging, Cell. (2011) 146, no. 5.

[35] Stahl E. C. , Haschak M. J. , Popovic B. , and Brown B. N. , Macrophages in the Aging Liver and Age-Related Liver Disease, Frontiers in Immunology. (2018) 9. 10.3389/fimmu.2018.02795 PMC6284020 30555477

[36] Yuxin X. , Qiuxiang L. , and Xuehua J. , Immunosenescence and Metabolic Reprogramming in MASLD: An Age-Dependent Immunometabolic Vicious Cycle and Therapeutic Opportunities, Frontiers in Cell and Developmental Biology. (2025) 13. 10.3389/fcell.2025.1650677 PMC12540461 41133219

[37] Gardner J. K. , Mamotte C. D. , McGonigle T. , Dye D. E. , Jackaman C. , and Nelson D. J. , Lipid-Laden Partially-Activated Plasmacytoid and CD4(-)CD8α(+) Dendritic Cells Accumulate in Tissues in Elderly Mice, Immunity & Ageing. (2014) 11. 10.1186/1742-4933-11-11 PMC4118209 25089147

[38] Agrawal A. , Sridharan A. , Prakash S. , and Agrawal H. , Dendritic Cells and Aging: Consequences for Autoimmunity, Expert Review of Clinical Immunology. (2011) 8. 10.1586/eci.11.77 PMC3285507 22149342

[39] Parker G. A. and Picut C. A. , Toxicologic Pathology, Liver Immunobiology. (2005) 33. 10.1080/01926230590522365 15805056

[40] Sato K. , Ohtsuka K. , and Hasegawa K. , et al.Evidence for Extrathymic Generation of Intermediate T Cell Receptor Cells in the Liver Revealed in Thymectomized, Irradiated Mice Subjected to Bone Marrow Transplantation, Journal of Experimental Medicine. (1995) 182, no. 3, 759–767, 10.1084/jem.182.3.759. 7650483 PMC2192177

[41] Connie J. , Federica T. , and Lelinh D. , et al.Aging and Cancer: The Role of Macrophages and Neutrophils, Ageing Research Reviews. (2017) 36. 10.1016/j.arr.2017.03.008 28390891

[42] Okuyama R. , Abo T. , and Seki S. , et al.Estrogen Administration Activates Extrathymic T Cell Differentiation in the Liver, Journal of Experimental Medicine. (1992) 175, no. 3, 661–669, 10.1084/jem.175.3.661. 1531494 PMC2119148

[43] Gibson K. L. , Wu Y.-C. , and Barnett Y. , et al.B-Cell Diversity Decreases in Old Age and Is Correlated With Poor Health Status, Aging Cell. (2008) 8, no. 1. 10.1111/j.1474-9726.2008.00443.x PMC2667647 18986373

[44] Daniela F. , Marie L. A. , and Suzanne C. L. , et al.Aging Down-Regulates the Transcription Factor E2A, Activation-Induced Cytidine Deaminase, and Ig Class Switch in Human B Cells, Journal of Immunology. (2008) 180, no. 8. 10.4049/jimmunol.180.8.5283 18390709

[45] Daniela F. , Alain D. , Maria R. , Marie L. A. , and Bonnie B. B. , Age Effects on B Cells and Humoral Immunity in Humans, Ageing Research Reviews. (2010) 10, no. 3. 10.1016/j.arr.2010.08.004 PMC3040253 20728581

[46] Saad K. , Mainak C. , and Fei W. , et al.B Cells Promote T Cell Immunosenescence and Mammalian Aging Parameters, 2024, bioRxiv.

[47] Nazish S. , Yingxiang H. , and Khiem N. , et al.An Inflammatory Aging Clock (iAge) Based on Deep Learning Tracks Multimorbidity, Immunosenescence, Frailty and Cardiovascular Aging, Nature Aging. (2021) 1. 10.1038/s43587-021-00082-y PMC8654267 34888528

[48] Raquel M.-D. , Martí O.-R. , and Anabel F.-I. , et al.Effects of Aging on Liver Microcirculatory Function and Sinusoidal Phenotype, Aging Cell. (2018) 17, no. 6. 10.1111/acel.12829 PMC6260924 30260562

[49] Michael A. P. , Irene A. , and Melissa K. , et al.A Cross-Sectional Study of Functional and Metabolic Changes During Aging Through the Lifespan in Male Mice, Elife. (2021) 10. 10.7554/eLife.62952 PMC8099423 33876723

[50] Ricardo M. and Carla C.-G. , Long Live the Liver: Immunohistochemical and Stereological Study of Hepatocytes, Liver Sinusoidal Endothelial Cells, Kupffer Cells and Hepatic Stellate Cells of Male and Female Rats Throughout Ageing, Cell and Tissue Research. (2016) 366, no. 3. 10.1007/s00441-016-2490-y 27595150

[51] Cogger V. C. , Warren A. , Fraser R. , Ngu M. , McLean A. J. , and Couteur D. G. L. , Preliminary Analysis of the Sinusoidal Endothelium and Space of Disse in Ageing Papio Hamadrayas, Comparative Hepatology. (2004) 3. 10.1186/1476-5926-2-S1-S26 PMC2410245 14960178

[52] Laurent G. , Nicole W. , and Alexander E. , et al.Defined p16(High) Senescent Cell Types Are Indispensable for Mouse Healthspan, Cell Metabolism. (2020) 32, no. 1. 10.1016/j.cmet.2020.05.002 32485135

[53] McLean A. J. , Cogger V. C. , and Chong G. C. , et al.Age-Related Pseudocapillarization of the Human Liver, The Journal of pathology. (2003) 200, no. 1, 112–7, 10.1002/path.1328. 12692849

[54] LeCouteur D. G. , Cogger V. C. , and McCuskey R. S. , et al.Age-Related Changes in the Liver Sinusoidal Endothelium: A Mechanism for Dyslipidemia, Annals of the New York Academy of Sciences. (2007) 1114, no. 1. 10.1196/annals.1396.003 17804522

[55] Knock D. L. and Brouwer A. , A B: Kupffer Cells and the Acute Phase Response: the Effect of Aging, Immunological Investigations. (1989) 18. 10.3109/08820138909112247 2499535

[56] Tetsu S. , Kenta J. , and Konomi O. , et al.Long-Term Administration of pDC-Stimulative *Lactococcus lactis* Strain Decelerates Senescence and Prolongs the Lifespan of Mice, International Immunopharmacology. (2018) 58. 10.1016/j.intimp.2018.03.024 29605632

[57] Kazuki N. , Kenji K. , Shuichi S. , and Yuji N. , Pit Cells as Liver-Associated Natural Killer Cells: Morphology and Function, Medical Electron Microscopy. (2004) 33, no. 1. 10.1007/s00795-003-0229-9 15057602

[58] Kimura M. , Watanabe H. , Sato S. , and Abo T. , et al.Female Predominance of Extrathymic T Cells in Mice: Statistical Analysis, Immunology Letters. (1994) 39, no. 3. 10.1016/0165-2478(94)90167-8 8034341

[59] Novobrantseva T. I. , Majeau G. R. , and Amatucci A. , et al.Attenuated Liver Fibrosis in the Absence of B Cells, Journal of Clinical Investigation. (2005) 115, no. 11. 10.1172/JCI24798 PMC1265860 16276416

[60] Warren A. , Cogger V. C. , Fraser R. , DeLeve L. D. , McCuskey R. S. , and Couteur D. G. L. , The Effects of Old Age on Hepatic Stellate C**ell** **s** , Current Gerontology and Geriatrics Research. (2011) 2011. 10.1155/2011/439835 PMC3114409 21687587

[61] Guogang X. , Songhua Q. , and Joseph S. , et al.Mitochondrial ACSS1-K635 Acetylation Knock-in Mice Exhibit Altered Metabolism, Cell Senescence, and Nonalcoholic Fatty Liver Disease, Science Advances. (2024) 10, no. 20. 10.1126/sciadv.adj5942 PMC11100568 38758779

[62] Anthony L. , Jack L. , and Marie-Helene R.-S. , et al.Neutrophils Induce Paracrine Telomere Dysfunction and Senescence in ROS-Dependent Manner, The EMBO Journal. (2021) 40, no. 9. 10.15252/embj.2020106048 PMC8090854 33764576

[63] Thomas G. B. , Miryam M. , and Luke B. , et al.TGFβ Inhibition Restores a Regenerative Response in Acute Liver Injury by Suppressing Paracrine Senescence, Science Translational Medicine. (2018) 10, no. 454. 10.1126/scitranslmed.aan1230 PMC6420144 30111642

[64] Yiji C. , Xue W. , and Bei W. , et al.Aging-Associated Oxidative Stress Inhibits Liver Progenitor Cell Activation in Mice, Aging. (2017) 9, no. 5. 10.18632/aging.101232 PMC5472737 28458256

[65] James F. , David J. , and Julia L. N. , Chronic Liver Disease in an Ageing Population, Age Ageing. (2008) 38, no. 1. 10.1093/ageing/afn242 19029099

[66] Le Couteur D. G. , Blyth F. M. , and Creasey H. M. , et al.The Association of Alanine Transaminase With Aging, Frailty, and Mortality, The Journals of Gerontology. Series A, Biological Sciences and Medical Sciences. (2010) 65, no. 7, 712–7, 10.1093/gerona/glq082. 20498223 PMC4085878

[67] Fleming K. M. , West J. , Aithal G. P. , and Fletcher A. E. , Abnormal Liver Tests in People Aged 75 and Above: Prevalence and Association With Mortality, Alimentary Pharmacology & Therapeutics. (2011) 34, no. 3. 10.1111/j.1365-2036.2011.04718.x 21631558

[68] Raquel M.-D. and Jordi G.-S. , Aging and Chronic Liver Disease, Seminars in Liver Disease. (2021) 40. 10.1055/s-0040-1715446 33764489

[69] David F. , Judith C. , and Eric V. , et al.Chronic Inflammation in the Etiology of Disease Across the Life Span, Nature Medicine. (2019) 25, no. 12. 10.1038/s41591-019-0675-0 PMC7147972 31806905

[70] Dominique T. , Sophie L. C. , and Vincent T. , et al.Hepatitis C in 6,865 Patients 65 yr or Older: A Severe and Neglected Curable Disease?, American Journal of Gastroenterology. (2006) 101, no. 6. 10.1111/j.1572-0241.2006.00556.x 16771947

[71] Zobair M. Y. , Aaron B. K. , Dinan A. , Yousef F. , Linda H. , and Mark W. , Global Epidemiology of Nonalcoholic Fatty Liver Disease-Meta-Analytic Assessment of Prevalence, Incidence, and Outcomes, Hepatology. (2015) 64, no. 1. 10.1002/hep.28431 26707365

[72] Yuichiro E. , Hideyuki H. , and Masafumi O. , et al.Prevalence and Associated Metabolic Factors of Nonalcoholic Fatty Liver Disease in the General Population From 2009 to 2010 in Japan: A Multicenter Large Retrospective Study, Journal of Gastroenterology. (2012) 47, no. 5. 10.1007/s00535-012-0533-z 22328022

[73] Aloysious A. , Cinzia S. , and Phaedra T. , et al.Hepatocyte Senescence Predicts Progression in Non-Alcohol-Related Fatty Liver Disease, Journal of Hepatology. (2012) 58, no. 3. 10.1016/j.jhep.2012.10.031 23142622

[74] Muhammad A. and Mark J. C. , Autophagy in Nonalcoholic Steatohepatitis, Expert Review of Gastroenterology & Hepatology. (2011) 5, no. 2. 10.1586/egh.11.4 PMC3104297 21476911

[75] Jingling J. , Polina I. , and Meghan B. , et al.Increased Expression of Enzymes of Triglyceride Synthesis Is Essential for the Development of Hepatic Steatosis, Cell Reports. (2013) 3, no. 3. 10.1016/j.celrep.2013.02.009 PMC3615099 23499441

[76] Cieslak K. P. , Baur O. , Verheij J. , Bennink R. J. , and van Gulik T. M. , Liver Function Declines With Increased Age, HPB. (2016) 18, no. 8. 10.1016/j.hpb.2016.05.011 PMC4972366 27485064

[77] Hisao W. , Yoshihiro N. , Takafumi U. , Takashi M. , and Hajime M. , Evaluation of the Effect of Age on Functioning Hepatocyte Mass and Liver Blood Flow Using Liver Scintigraphy in Preoperative Estimations for Surgical Patients: Comparison With CT Volumetry, Journal of Surgical Research. (2002) 106, no. 2. 10.1006/jsre.2002.6462 12175974

[78] Sotaniemi E. A. , Arranto A. J. , Pelkonen O. , and Pasanen M. , Age and Cytochrome P450-Linked Drug Metabolism in Humans: An Analysis of 226 Subjects With Equal Histopathologic Conditions, Clinical Pharmacology & Therapeutics. (1997) 61, no. 3, 331–339, 10.1016/S0009-9236(97)90166-1. 9091249

[79] Chalasani N. , Bonkovsky H. L. , and Fontana R. , et al.Features and Outcomes of 899 Patients With Drug-Induced Liver Injury: The DILIN Prospective Study, Gastroenterology. (2015) 148, no. 7. 10.1053/j.gastro.2015.03.006 PMC4446235 25754159

[80] Lucena M. I. , Sanabria J. , Stephens C. , Andrade R. J. , and García-Cortes M. , Drug-Induced Liver Injury in Older People, The Lancet Gastroenterology & Hepatology. (2020) 5, no. 9. 10.1016/S2468-1253(20)30006-6 32818465

[81] Lucena M. I. , Andrade R. J. , and Kaplowitz N. , et al.Phenotypic Characterization of Idiosyncratic Drug-Induced Liver Injury: The Influence of Age and Sex, Hepatology. (2009) 49, no. 6. 10.1002/hep.22895 19475693

[82] Andrade R. J. , Chalasani N. , and Björnsson E. S. , et al.Drug-Induced Liver Injury, Nature Reviews Disease Primers. (2019) 5, no. 1. 10.1038/s41572-019-0105-0 31439850

[83] Lily D. , Zhang-Xu L. , and Neil K. , Mechanisms of Adaptation and Progression in Idiosyncratic Drug Induced Liver Injury, Clinical Implications, Liver International. (2015) 36, no. 2. 10.1111/liv.12988 PMC4718752 26484420

[84] Sadighi Akha A. A. , Aging and the Immune System: An Overview, Journal of Immunological Methods. (2018) 463, 21–26, 10.1016/j.jim.2018.08.005. 30114401

[85] Jay H. H. and Einar S. B. , Drug-Induced Liver Injury - Types and Phenotypes, The New England Journal of Medicine. (2019) 381, no. 3. 10.1056/NEJMra1816149 31314970

[86] OToole P. W. and Jeffery I. B. , Gut Microbiota and Aging, Science. (2016) 350, no. 6265. 10.1126/science.aac8469 26785481

[87] Branca J. J. V. , Gulisano M. , and Nicoletti C. , Intestinal Epithelial Barrier Functions in Ageing, Ageing Research Reviews. (2019) 50, 100938. 10.1016/j.arr.2019.100938 31369869

[88] Marwan G. , Jiezhun G. , and Lindsay Y. , et al.Development and Validation of a Model Consisting of Comorbidity Burden to Calculate Risk of Death Within 6 Months for Patients With Suspected Drug-Induced Liver Injury, Gastroenterology. (2019) 157, no. 5. 10.1053/j.gastro.2019.07.006 PMC6815697 31302142

[89] Macias R. I. R. , Monte M. J. , and Serrano M. A. , et al.Impact of Aging on Primary Liver Cancer: Epidemiology, Pathogenesis and Therapeutics, Aging. (2021) 13, no. 19, 23416–23434, 10.18632/aging.203620. 34633987 PMC8544321

[90] Couteur D. G. L. and Thillainadesan J. , et al.What Is an Aging-Related Disease? An Epidemiological Perspective, The Journals of Gerontology: Series A. (2022) 77, no. 11. 10.1093/gerona/glac039 PMC9678203 35167685

[91] Claudio F. , Paolo G. , Paolo P. , Cristina G. , and Aurelia S. , Inflammaging: A New Immune-Metabolic Viewpoint for Age-Related Diseases, Nature Reviews Endocrinology. (2018) 14, no. 10. 10.1038/s41574-018-0059-4 30046148

[92] Michael K. , New Insights Into the Pathogenesis and Treatment of Non-Viral Hepatocellular Carcinoma: A Balancing Act Between Immunosuppression and Immunosurveillance, Precision Clinical Medicine. (2019) 1, no. 1. 10.1093/pcmedi/pby005 PMC6333043 30687560

[93] Kirkland J. L. and Tchkonia T. , Senolytic Drugs: From Discovery to Translation, Journal of Internal Medicine. (2020) 288, no. 5, 518–536, 10.1111/joim.13141. 32686219 PMC7405395

